# More Severe Manifestations and Poorer Short-Term Prognosis of Ganglioside-Associated Guillain-Barré Syndrome in Northeast China

**DOI:** 10.1371/journal.pone.0104074

**Published:** 2014-08-01

**Authors:** Xiujuan Wu, Wei Wu, Zhengzheng Wang, Donghui Shen, Wei Pan, Ying Wang, Limin Wu, Xiaokun Wu, Jiachun Feng, Kangding Liu, Jie Zhu, Hong-Liang Zhang

**Affiliations:** 1 Neuroscience Center, Department of Neurology, the First Hospital of Jilin University, Jilin University, Changchun, China; 2 Department of Neurosurgery, the First Hospital of Jilin University, Jilin University, Changchun, China; 3 School of Public Health, Jilin University, Changchun, China; 4 Norman Bethune Health Science Center, Jilin University, Changchun, China; 5 Neuroprotection Research Laboratory, Massachusetts General Hospital, Harvard Medical School, Boston, Massachusetts, United States of America; 6 Department of Neurobiology, Care Sciences and Society, Karolinska Institute, Stockholm, Sweden; Oslo University Hospital, Norway

## Abstract

Ganglioside as a neurotrophic drug has been hitherto widely used in China, although Guillain-Barré syndrome (GBS) following intravenous ganglioside treatment was reported in Europe several decades ago. We identified 7 patients who developed GBS after intravenous use of gangliosides (ganglioside+ group) and compared their clinical data with those of 77 non-ganglioside-associated GBS patients (ganglioside− group) in 2013, aiming at gaining the distinct features of ganglioside-associated GBS. Although the mean age, protein levels in cerebrospinal fluid (CSF) and frequency of cranial nerve involvement were similar between the two groups, the Hughes Functional Grading Scale (HFGS) score and the Medical Research Council (MRC) sum score at nadir significantly differed (4.9±0.4 vs 3.6±1.0; 7.7±5.5 vs 36.9±14.5, both *p*<0.001), indicating a higher disease severity of ganglioside-associated GBS. A higher ratio of patients with ganglioside-associated GBS required mechanical ventilation (85.7% vs 15.6%, *p*<0.01). The short-term prognosis of ganglioside-associated GBS, as measured by the HFGS score and the MRC sum score at discharge, was poorer (4.3±0.5 vs 2.8±1.1; 17.3±12.9 vs 46.0±13.9, both *p*<0.001). All the patients in the ganglioside+ group presented an axonal form of GBS, namely acute motor axonal neuropathy (AMAN). When compared with the AMAN patients in the ganglioside− group, more severe functional deficits at nadir and poorer recovery after standard treatment were still prominent in ganglioside-associated GBS. Anti-GM1 and anti-GT1a antibodies were detectable in patients with AMAN while not in patients with the demyelinating subtype of GBS. The concentrations of these antibodies in patients with AMAN were insignificantly different between the ganglioside+ and ganglioside− groups. In sum, ganglioside-associated GBS may be a devastating side effect of intravenous use of gangliosides, which usually manifests a more severe clinical course and poorer outcome.

## Introduction

Guillain-Barré syndrome (GBS) is an immune-mediated disorder in the peripheral nervous system with inflammatory infiltration, demyelination and/or axonal damage as the main pathological features [1]. Depending on the involvement of nerve fiber types (sensory, motor and autonomic) and the predominant nature of nerve injury (demyelination or axonal degeneration), GBS can be divided into acute inflammatory demyelinating polyneuropathy (AIDP), acute motor axonal neuropathy (AMAN), acute motor-sensory axonal neuropathy (AMSAN), Miller-Fisher syndrome (MFS) and some other relatively rare subtypes [1]. AIDP and AMAN are the major subtypes of GBS and they are distinct from each other in terms of immunopathogenesis, electrophysiological findings, pathological changes and responses to treatment, etc [2]. AMAN, a pure motor axonal subtype of GBS, which was initially identified from GBS patients in northern China [3], is more frequent in eastern Asia and Central and South America [2]. Immunohistochemical studies on AMAN revealed antibody-mediated damage to the motor axonal membrane suggestive of an immune response directed against the motor axolemma [3]–[4]. AMASN is another axonal subtype of GBS in which axonal degeneration involves both motor and sensory nerves [1]. AMAN and AMASN are associated with *Campylobacter jejuni* enteritis and antibodies against gangliosides [2]. High titers of anti-GM1 antibodies were found in patients who developed GBS following exogenous gangliosides injection [5]–[7], leading to the suspicion that exogenous gangliosides might be foreign to humans and may act as an immunogenic agent. The animal model of AMAN shares pathological features with human AMAN [8]. Despite reports on GBS following intravenous use of gangliosides in Europe several decades ago, which led to its withdrawal from European market [9], ganglioside as a nutritional agent has been hitherto widely used in China and ganglioside-associated GBS cases have been rarely documented. Therefore, it remains unknown whether the clinical course and the outcome of ganglioside-associated GBS are distinguishable from non-ganglioside-associated sporadic GBS. In this study, we identified patients who developed GBS after receiving intravenous gangliosides and compared them with those without receiving gangliosides, aiming to depict a distinctive picture of ganglioside-associated GSB.

## Materials and Methods

### Study subjects

This study was approved by the ethics committee of the First Hospital of Jilin University, Changchun, China. Written informed consent was obtained from all patients. All GBS patients who were admitted to Department of Neurology of the First Hospital of Jilin University in 2013 were enrolled. These patients fulfilled the diagnostic criteria for GBS [10]. The Department of Neurology of the First Hospital of Jilin University is the largest center for the diagnosis and treatment of neurological diseases in northeast China. Gangliosides as an exclusive component (monosialotetrahexosylganglioside sodium salt injection) or part of a compound (cattle encephalon glycoside and ignotin injection) have never been used in our department and all the enrolled patients were referred to our department from other departments or from other hospitals. Patients diagnosed as MFS or chronic inflammatory demyelinating polyneuropathy (CIDP) were ruled out. Critical illness polyneuropathy as the most common cause of acute flaccid paralysis in hospital was also excluded [11]. All these ganglioside-associated GBS patients received gangliosides intravenously for treating other diseases while subsequently developed fulminant acute polyneuropathy during or after the treatment.

### Grouping and treatment

Enrolled subjects were divided into the ganglioside+ group (ganglioside-associated) and the ganglioside− group (non-ganglioside-associated) according to whether they received exogenous gangliosides before disease onset. Diagnosis of AIDP or AMAN was based on the electrophysiological criteria proposed by Hadden and colleagues [12].

All patients received a standard treatment with intravenous immunoglobulin (IVIG, 0.4 g/kg body weight per day, for 5 consecutive days), immediately clinical diagnosis was established after admission. Patients whose functional deficits kept deteriorating despite the use of IVIG were treated with corticosteroids (pulse methylprednisolone 1000 mg for 3 days and gradually tapered).

### Evaluation of clinical severity and functional impairment

The clinical severity and functional impairment were evaluated for all the enrolled GBS subjects. Motor function deficits of patients were scored by the Hughes Functional Grading Scale (HFGS) score ranging from 0 to 6. The scale was specifically defined as follows: 0: healthy state; 1: minor symptoms and capable of running; 2: able to walk 5 m or more without assistance but unable to run; 3: able to walk 5 m across an open space with help; 4: bedridden or chair bound; 5: requiring assisted ventilation for at least part of the day; 6: dead [13]. Neurologic function was also evaluated by using the Medical Research Council (MRC) sum score of six bilateral muscles in arms and legs, ranging from 0 (tetraparalytic) to 60 (normal strength) [14]. Nadir of the disease was defined as the highest HFGS score or the lowest MRC sum score.

### CSF and plasma sample collection

Samples of CSF were obtained from lumbar puncture and plasma from venous puncture after an informed consent was acquired. CSF samples that appeared turbid or mixed with blood were excluded. CSF and plasma samples were then aliquoted and stored at −80°C until further analysis.

### ELISA for measurement of anti-ganglioside (GM1 and GT1a) antibodies

Paired samples of CSF and plasma were acquired from patients with GBS. ELISA kits for detection of anti-GM1 antibodies and anti-GT1a IgG antibodies were purchased from manufacturers (R&D Systems, Minneapolis, MN, US), and detecting procedures were followed according to their instructions. The kits applied the quantitative sandwich enzyme immunoassay technique. Microtiter plates had been pre-coated with ganglioside antigen (GM1 or GT1a). Standards or samples were then added to the microtiter plate wells and ganglioside antibodies would bind to the antigen pre-coated wells. A standardized preparation of horseradish peroxidase-conjugated ganglioside antigen was added to “sandwich” the ganglioside antibodies (anti-GM1 or anti-GT1a) immobilized on the plate. The microtiter plates underwent 2 h incubation, and then the wells were thoroughly washed to remove all unbound components. Substrate solutions were then added to each well. The enzyme (horseradish peroxidase) and substrate were allowed to react over a short incubation period (30 min). The reaction was terminated by adding a sulphuric acid solution and the color change was measured spectrophotometrically at a wavelength of 450 nm by an ELISA reader. A standard curve was plotted relating the intensity of the color (O.D.) to the concentration of the standards. The concentrations of ganglioside antibodies in each sample were extrapolated from this standard curve.

### Statistical analysis

Statistical analysis was performed with SPSS version 17.0 software (SPSS, IBM, West Grove, PA, USA). Data were expressed as mean ± standard deviation. The Chi-square or Fisher exact tests were used for discrete variables and the student-*t* tests for continuous variables. For all statistical tests, *p*<0.05 was considered significant.

## Results

### Demographic features of patients with ganglioside-associated GBS

From January 1 to December 31, 2013, a total of 84 patients with GBS, aged from 18 to 84 years old, were consecutively enrolled in this study, among whom 58.3% were males. Subjects were divided into the ganglioside+ group and the ganglioside− group. Seven patients fell into the ganglioside+ group and the detailed clinical features are shown in Table 1. Five (71.4%) of them were males with the mean age of 50.

**Table 1:** Demographic data of patients with ganglioside-associated GBS.

	Case 1	Case 2	Case 3	Case 4	Case 5	Case 6	Case 7
**Sex**	Female	Male	Male	Male	Male	Male	Female
**Age**	47 y	31 y	40 y	77 y	58 y	41 y	60 y
**Primary disease**	Cerebral hemorrhage	Cerebral hemorrhage and SAH	Head trauma	Vertebral fracture	Renal contusion	AVM	Lacunar infarction
**Ganglioside therapy duration**	14 d	7 d	9 d	7 d	5 d	7 d	7 d
**Cranial nerve involvement**	Bilateral facial nerve palsy	Bilateral facial nerve palsy	Spared	Spared	Spared	Spared	Spared
**HFGS score at nadir**	5	5	5	5	5	4	5
**MRC sum score at nadir**	12	4	4	5	8	18	3
**Ventilation**	Yes	Yes	Yes	Yes	Yes	No	Yes
**EMG findings**	Axonal impairment	Axonal impairment	Mainly axonal impairment	Axonal impairment	Axonal impairment	Axonal impairment	Axonal impairment
**Lumbar puncture**	Done	Done	Undone	Done	Done	Done	Undone
**AD in CSF**	Yes	Yes	N/A	Yes	No	Yes	N/A
**Protein level of CSF (g/L)**	2.23	0.67	N/A	1.09	0.35	1.26	N/A
**Treatment**	IVIG+G	IVIG+G	IVIG	IVIG+G	IVIG+G	IVIG	IVIG
**Hospital stay**	62 d	90 d	2 d	96 d	82 d	34 d	55 d
**HFGS score at discharge**	4	4	5	5	4	4	4
**MRC sum score at discharge**	28	8	4	5	34	30	12

GBS: Guillain-Barré syndrome; SAH: subarachnoid hemorrhage; AVM: arteriovenous malformation; HFGS: the Hughes Functional Grading Scale; MRC: the Medical Research Council; EMG: electromyography; AD: albuminocytologic dissociation; CSF: cerebrospinal fluid; IVIG: intravenous immunoglobulin; G: glucocorticoids.

**Figure 1:**
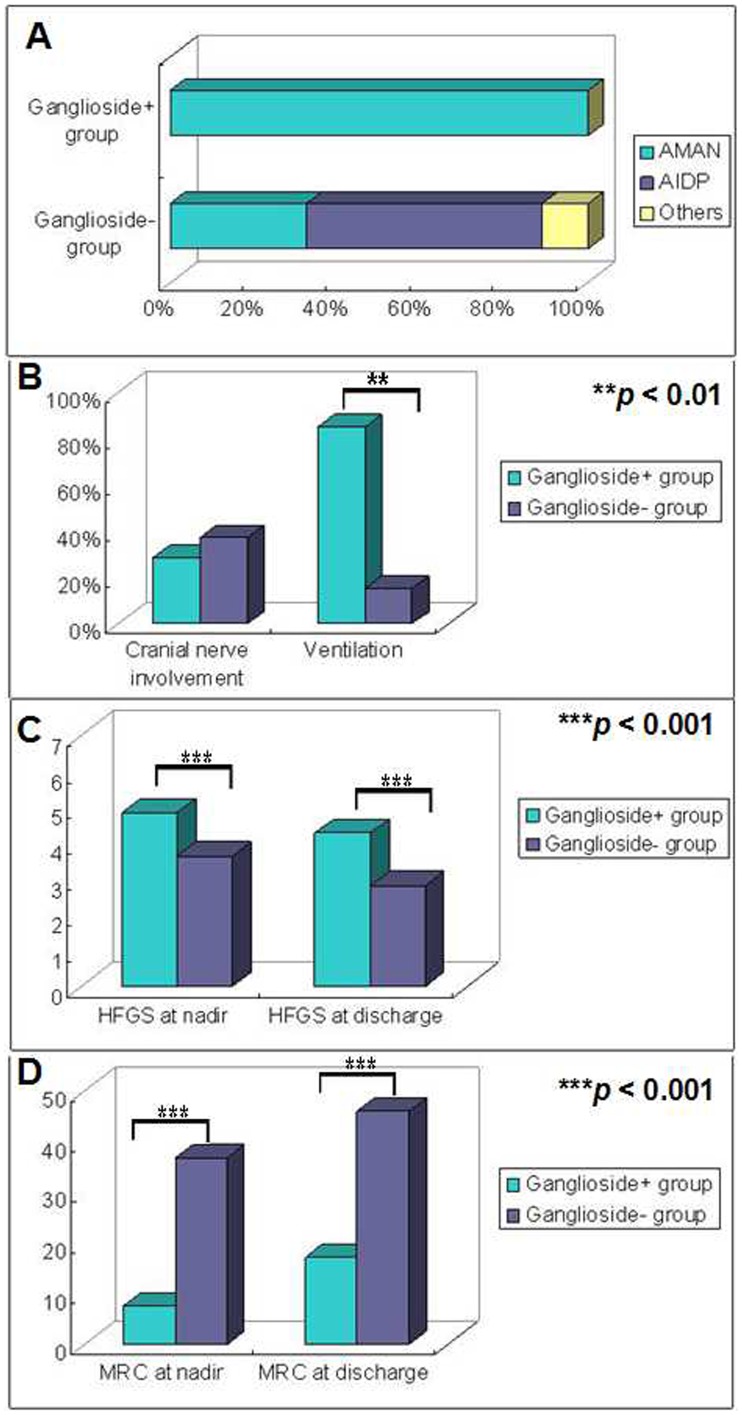
Comparisons of electrophysiological findings and functional deficits between the ganglioside+ and ganglioside− groups. **A**. Electrophysiological examinations on patients in the ganglioside+ group revealed impairment mainly to axons suggestive of acute motor axonal neuropathy (AMAN). In the non-ganglioside-associated group (ganglioside− group), 46 out of 77 patients (59.7%) received electrophysiological examinations, and the ratios of acute inflammatory demyelinating polyneuropathy (AIDP) and AMAN were 56.5% and 32.6%, respectively. **B**. Two patients (28.6%) in the ganglioside+ group while 29 (37.7%) in the ganglioside− group were found with bilateral facial nerves palsy (*p*>0.05). The difference did not reach statistical significance. Ventilation ratio was significantly higher in the ganglioside+ group as compared that in the ganglioside− group (*p*<0.01). **C**. The scores of Hughes Functional Grading Scale (HFGS) were significantly different between the ganglioside+ group and the ganglioside− group, both at nadir and at discharge, and higher HFGS scores were found in the ganglioside+ group (*p*<0.001), suggestive of severe clinical course and poor short-term outcome. **D**. The Medical Research Council sum scores (MRC) were significantly lower in the ganglioside+ group both at nadir and at discharge (*p*<0.001).

In the ganglioside+ group, patients developed flaccid paralysis during or several days after ganglioside treatment for other diseases without any antecedent infections. The interval from the first use of gangliosides to the onset of polyneuropathy ranged from 5 to 14 days. Six patients (85.7%) developed respiratory muscle paralysis requiring intubation and ventilation. Two patients (28.6%) had bilateral facial nerves palsy. Lumbar puncture was performed in 5 patients and albumino-cytologic dissociation of CSF was found in 4 of them (80%). Electrophysiological examinations revealed mainly the impairment of axons suggestive of AMAN (Figure 1A). The HFGS score and the MRC sum score were 4.9±0.4 and 7.7±5.5, respectively, at nadir, while they were 4.3±0.5 and 17.3±12.9, respectively, at discharge.

Seventy-seven subjects fell into the ganglioside− group with the mean age of 45. Forty-two (54.6%) of them were males. In the ganglioside− group, 50 (64.9%) patients developed polyneuropathy out of hospitals with antecedent respiratory or gastrointestinal infections. Use of ganglioside was not identified in these patients. Respiratory muscle paralysis requiring intubation and ventilation was found in 12 patients (15.6%) and cranial nerve involvement was found in 29 patients (37.7%). Lumbar puncture was performed in 63 patients. Albumino-cytologic dissociation of CSF was found in 41 patients (65.1%) while normal protein concentrations in CSF were found in 8 patients (12.7%). Interestingly, slight pleocytosis with increased concentrations of protein was found in 14 patients (22.2%). Electrophysiological examination was performed in 46 patients (59.7%). The distribution of the subtypes of GBS in the ganglioside− group is showed in Figure 1A. The HFGS score and the MRC sum score were 3.6±1.0 and 36.9±14.5 respectively at nadir while were 2.8±1.1 and 46.0±13.9 respectively at discharge.

### More severe clinical course and poorer short-term prognosis of ganglioside-associated GBS

The comparisons of the clinical features between the two groups are showed in Table 2. The age and cranial nerve involvement as showed in Figure 1B, and protein concentrations in CSF of the two groups were not different. Higher HFGS and lower MRC scores were noted in the ganglioside+ group both at nadir and at discharge (Figure 1C and D), suggestive of a severe clinical course and poor outcome in the ganglioside+ group. Additionally, respiratory muscle paralysis requiring intubation and ventilation was more frequent in the ganglioside+ group, which was shown in Figure 1B.

**Table 2:** Comparisons of clinical features between patients with ganglioside-associated GBS and non-ganglioside-associated GBS.

Variables	Ganglioside+ group	Ganglioside− group	*p*-value
**Age (year-old)**	50.6±15.0	45.4±15.6	>0.05
**AMAN**	100% (7/7)	32.6% (15/46)	<0.01
**HFGS score at nadir**	4.9±0.4	3.6±1.0	<0.001
**MRC sum score at nadir**	7.7±5.5	36.9±14.5	<0.001
**Ventilation**	85.7% (6/7)	15.6% (12/77)	= 0.001 <0.01
**Cranial nerve involvement**	28.6% (2/7)	37.7% (29/77)	>0.05
**Protein level of CSF (g/L)**	1.1±0.7	1.2±1.1	>0.05
**HFGS score at discharge**	4.3±0.5	2.8±1.1	<0.001
**MRC sum score at discharge**	17.3±12.9	46.0±13.9	<0.001

GBS: Guillain-Barré syndrome; AMAN: acute motor axonal neuropathy; CSF: cerebrospinal fluid; HFGS: the Hughes Functional Grading Scale; MRC: the Medical Research Council.

### More severe clinical course and poorer short-term prognosis of ganglioside-associated AMAN compared with non-ganglioside-associated AMAN

**Figure 2:**
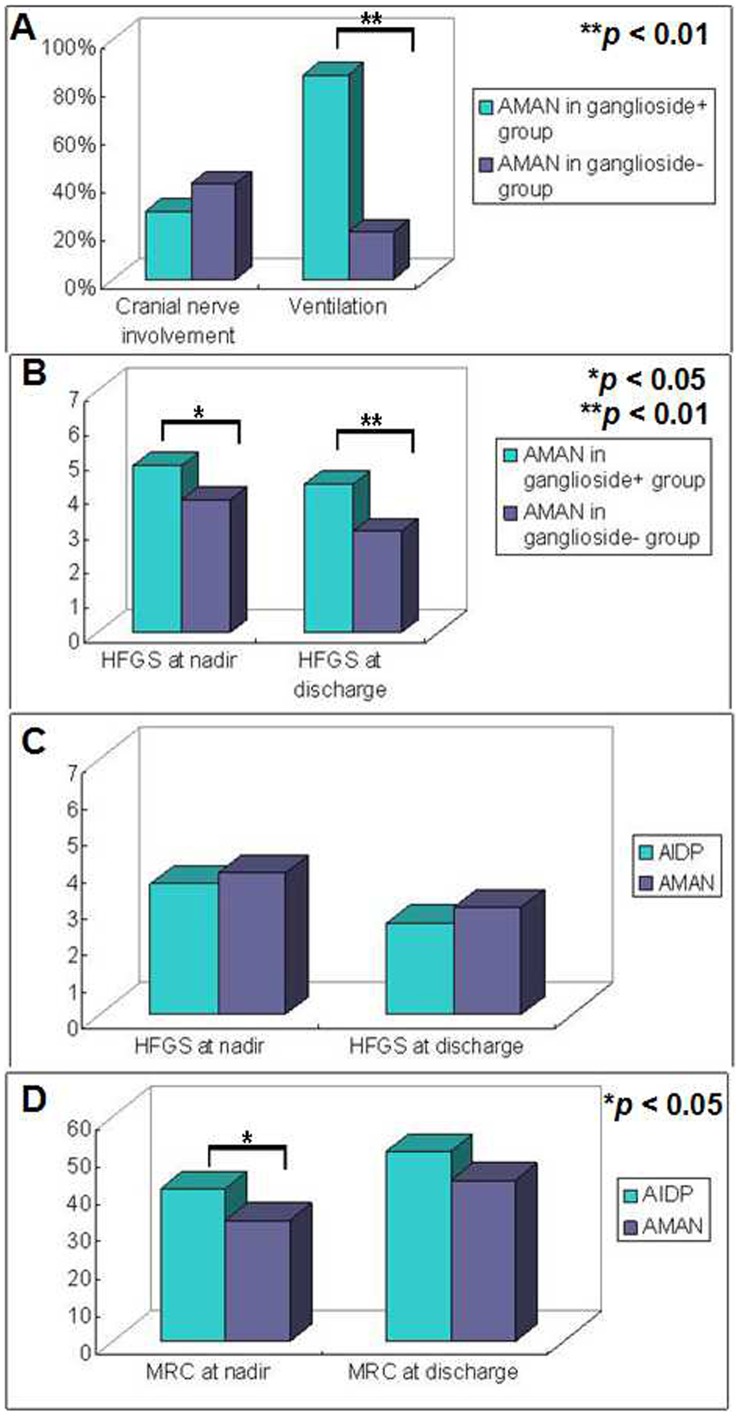
Stratified analyses of electrophysiological findings and functional deficits in patients according to subtypes (axonal form or demyelinating form) between the ganglioside+ and ganglioside− groups. **A**. Two acute motor axonal neuropathy (AMAN) patients (28.6%) in the ganglioside+ group while 6 out of 15 (40%) in the ganglioside− group were found with bilateral facial nerves palsy. However, the frequency of cranial nerve involvement did not significantly differ in AMAN patients between the ganglioside+ group and ganglioside− group. The ventilation ratio was significantly higher in the ganglioside+ group when compared with that in the ganglioside− group (*p*<0.01). **B**. The scores of Hughes Functional Grading Scale (HFGS) were higher in AMAN patients in the ganglioside+ group both at nadir and at discharge suggestive of more severe clinical course and poorer short-term outcome. **C**. The scores of HFGS at nadir and at discharge were insignificantly different between patients with acute inflammatory demyelinating polyneuropathy (AIDP) and AMAN in the ganglioside− group (both *p*>0.05). **D**. The sum score of Medical Research Council (MRC) at nadir was significantly higher in patients with AIDP in the ganglioside− group than AMAN (*p*<0.05).

Given the fact that all patients presented an axonal form of GBS, i.e. AMAN in the ganglioside+ group, we further compared the clinical features between the AMAN patients in the ganglioside+ group and in the ganglioside− group. The results are shown in Table 3. The age, the ratio of cranial nerve involvement and the protein level in CSF were insignificantly different between the two groups (Figure 2A). The scores of HFGS at nadir and at discharge of patients with AMAN (Figure 2B), significantly differed between the two groups, indicating a severe clinical course of AMAN patients following ganglioside treatment. Likewise, the MRC sum scores at nadir and at discharge were different between the two groups as well suggestive of a severe clinical course and poor outcome of AMAN in the ganglioside+ group. Additionally, respiratory muscle paralysis requiring intubation and ventilation was more frequent in the ganglioside+ group (Figure 2A). The mean hospitalization duration of the AMAN patients was 60 and 22 days in the ganglioside+ group and in the ganglioside− group, respectively.

**Table 3:** Comparisons of clinical features of AMAN patients between the ganglioside+ group and the ganglioside− group.

Variables	Ganglioside+ group (7/7)	Ganglioside− group (15/77)	*p*-value
**Age (year-old)**	50.6±15.0	48.3±16.4	>0.05
**HFGS score at nadir**	4.9±0.4	3.9±0.9	= 0.013 <0.05
**MRC sum score at nadir**	7.7±5.5	32.3±16.5	= 0.001 <0.01
**Ventilation ratio**	85.7% (6/7)	20% (3/15)	= 0.007 <0.01
**Cranial nerve involvement**	28.6% (2/7)	40% (6/15)	>0.05
**Protein level in CSF (g/L)**	1.1±0.7	1.1±0.6	>0.05
**HFGS score at discharge**	4.3±0.5	2.9±1.1	= 0.001 <0.01
**MRC sum score at discharge**	17.3±12.9	43.0±14.9	= 0.001 <0.01

AMAN: acute motor axonal neuropathy; HFGS: the Hughes Functional Grading Scale; MRC: the Medical Research Council; CSF: cerebrospinal fluid.

### Comparisons between AIDP and AMAN patients in the ganglioside− group

Forty-six patients received electrophysiological examinations to identify the clinical subtypes and 41 patients could be categorized as either AIDP or AMAN. The clinical features of AIDP and AMAN in the ganglioside− group are shown in Table 4. The age, the frequency of cranial nerve involvement, the ventilation ratio and the concentrations of protein in CSF were similar between the two groups. The HFGS score and the MRC sum score were displayed in Figure 2C and D. The MRC sum score at nadir was significantly higher in patients with AIDP than in the patients with AMAN.

**Table 4:** Comparisons of clinical features between AIDP and AMAN patients in the ganglioside− group.

Variables	AIDP (26)	AMAN (15)	*p*-value
**Age (year-old)**	44.6±11.6	48.3±16.4	>0.05
**HFGS score at nadir**	3.6±1.0	3.9±0.9	>0.05
**MRC sum score at nadir**	40.9±9.8	32.3±16.5	= 0.042 <0.05
**Ventilation ratio**	15.4% (4/26)	20% (3/15)	>0.05
**Electrophysiology**	Demyelination	Axonal damage	N/A
**Cranial nerve involvement**	38.5% (10/26)	40% (6/15)	>0.05
**Protein level in CSF (g/L)**	1.1±1.2	1.1±0.6	>0.05
**HFGS score at discharge**	2.5±1.0	2.9±1.1	>0.05
**MRC sum score at discharge**	50.7±7.08	43.0±14.9	>0.05

AIDP: acute inflammatory demyelinating polyneuropathy; AMAN: acute motor axonal neuropathy; HFGS: the Hughes Functional Grading Scale; MRC: the Medical Research Council; CSF: cerebrospinal fluid.

### Anti-ganglioside antibodies in CSF and plasma of AMAN patients

Anti-GM1 and anti-GT1a antibodies were detectable in AMAN patients. However, neither of them was detectable in AIDP patients. The concentrations of anti-GM1 antibodies of AMAN patients in the ganglioside+ group and in the ganglioside− group were 288.3±116.9 ng/L and 232.6±154.8 ng/L, respectively, in plasma (*p*>0.05), and 9.9±1.6 ng/L and 10.6±2.8 ng/L, respectively, in CSF (*p*>0.05). The concentrations of anti-GT1a antibodies of AMAN patients in the ganglioside+ group and in the ganglioside− group were 320.2±157.5 ng/L and 238.7±130.7 ng/L, respectively, in plasma (*p*>0.05), and 34.7±7.8 ng/L and 21.3±4.6 ng/L, respectively, in CSF (*p*>0.05).

Correlation analysis revealed negative results between clinical severity scores and concentrations of anti-ganglioside antibodies in CSF and plasma of AMAN patients, both in the ganglioside+ group and in the ganglioside− group.

## Discussion

Gangliosides have long been intravenously used as a neurotrophic drug in China. Despite safety concern aroused in Europe several decades ago [9], intravenous use of gangliosides in China has been believed to be safe and GBS as a severe side effect of this drug has not been documented in literature. We herein reported a case series of 7 patients who developed GBS after intravenous use of gangliosides. All these patients presented an axonal form of GBS, remarkably higher severity and poorer short-term prognosis. The association between exogenous gangliosides and GBS was further evidenced by the positivity of anti-GM1 and anti-GT1a antibodies in CSF and plasma, the albumino-cytologic dissociation of CSF and the result of the electrophysiological examination.

Although rare, GBS may occur following noninfectious events. Among various noninfectious factors associated with GBS, the most interesting one is exogenous gangliosides [15]–[16]. It had been extensively prescribed in various countries mainly for the treatment of peripheral neuropathies because of its neurotrophic role in neural regeneration [15]. However, numerous cases of GBS after ganglioside treatment raised the suspicion that exogenous gangliosides may cause GBS [9], [16]. Clinical monitoring studies on the relationship between exogenous gangliosides and GBS were hence conducted. However, the relationship remains controversial [17]–[21].

Although the pathogenesis of GBS secondary to gangliosides treatment was incompletely clarified, high titers of anti-GM1 antibodies were found in some of the patients who developed GBS after receiving a ganglioside therapy, leading to the suspicion that exogenous gangliosides might be foreign to humans and might be neuritogenic in humans [5]–[7]. Molecular mimicry has been proposed to explain the pathogenesis of *Campylobacter jejuni* associated GBS with positive antibodies against self-gangliosides [22]–[23]. Therefore, we postulate that development of GBS following gangliosides treatment might share a homologous pathogenesis with *Campylobacter jejuni* associated GBS. In Odaka et al's study on antibodies to GBS following gangliosides therapy, they found that GM1 is the major immunogen [7]. Further absorption studies and secondary ion mass spectra revealed the possible role of gangliosides in ganglioside-associated GBS [7]. Yuki and colleagues succeeded in establishing an AMAN model by sensitizing Japanese white rabbits with a bovine brain ganglioside mixture [8]. These rabbits developed high titers of anti-GM1 IgG antibody [8]. Moreover, the pathological findings showed predominant Wallerian-like degeneration with neither lymphocytic infiltration nor demyelination which was similar to the pathological changes with AMAN [8].

It is noteworthy that gangliosides have been widely used in China for many years. However, there has been no report on development of GBS in China thus far. It remains unclear why the seven patients developed GBS after gangliosides treatment among numerous patients who received intravenous gangliosides. One explanation is that the individual's genetic background might contribute to the development of GBS. It is also likely that trace amounts of endotoxin, for instance, might contribute to the development of GBS by acting as adjuvants in these patients. Contamination with bacterial components has been reported in the production process of recombinant human heat shock proteins [24]–[25]. The less purified gangliosides might trigger GBS via alteration of individual's susceptibility in a way of “molecular mimicry”, and led to the occurrence of disease [22]–[23]. Since GBS is an immune-mediated disorder [1], altered immune function resulting from various diseases in the 7 patients of the ganglioside+ group may play a synergistic role in the pathogenesis of GBS. Quality control for ganglioside production and intensified monitoring for side effects are therefore strongly suggested for the safe use of gangliosides in China.

Limitations of our study include the relatively small sample size, the failure to identify the subtypes of anti-ganglioside antibodies and the retrospective nature of data collection. The functional scores as measured with HFGS and the MRC sum score might be confounded by the motor deficits before the onset of GBS. However, this first-ever reported case series of ganglioside-assoicated GBS in China may alert us to this devastating side effect of intravenous use of gangliosides. Further studies are warranted to clarify the underlying pathogenesis of ganglioside-assoicated GBS.

In sum, exogenous gangliosides may be associated with development of GBS due to incompletely recognized pathogenesis. Ganglioside-associated GBS is more severe in clinical course with poorer short-term prognosis as compared with non-ganglioside-associated GBS in northeast China.
